# Mobile App–Guided Exposure Therapy for Panic Disorder With and Without Agoraphobia: Randomized Controlled Trial

**DOI:** 10.2196/76389

**Published:** 2025-11-19

**Authors:** Matthias Guth, Annika Wiebe, Mogda Ekhlas-Schumann, Alexa Rodenjohann, Felicia Rohlfsen, Jacqueline Buchholz, Benjamin Selaskowski, Alexandra Philipsen, Niclas Braun

**Affiliations:** 1 Department of Psychiatry and Psychotherapy, University Hospital Bonn, Venusberg-Campus 1, Bonn, 53127, Germany, +49-228-287-15723

**Keywords:** panic disorder, agoraphobia, mobile intervention, digital health, mobile health, exposure therapy

## Abstract

**Background:**

Mobile apps that implement disorder-specific psychotherapy for panic disorder (PD) with and without agoraphobia can be used in real-life situations that trigger symptoms and, therefore, are a promising novel therapeutic tool.

**Objective:**

This randomized controlled trial aimed to expand the currently limited evidence base for the efficacy of mobile interventions for PD with and without agoraphobia by evaluating a mobile app focusing on interoceptive and in-vivo exposure therapy.

**Methods:**

After establishing the diagnosis of PD with and without agoraphobia using a secure video communication platform, we randomized 111 adults to 3 equally sized groups: disorder-specific exposure therapy app, mindfulness meditation app unrelated to the disorder (active control condition), and waiting list (passive control condition). Participants used the apps in a self-guided manner. Additional psychotherapy was not allowed during the study. Self-reported PD with and without agoraphobia symptom severity was our primary outcome parameter and measured by the Panic and Agoraphobia Scale and symptom subset scales. Secondary outcome parameters included depressive symptoms and quality of life. We conducted assessments at baseline, after the intervention (allocation+5 wk), and at follow-up (allocation+13 wk).

**Results:**

We observed significantly lower overall PD with and without agoraphobia symptom severity in the exposure app group compared to waiting list post-treatment (*P*=.04, Cohen *d*=0.55) and at follow-up (*P*=.04, Cohen *d*=0.60). At follow-up, the exposure app group demonstrated significantly stronger improvements than the waiting list group in depressive symptoms (*P*=.007, Cohen *d*=0.75) and psychological quality of life (*P*=.01, Cohen *d*=0.63). We observed no significant differences between the exposure and the meditation apps in overall PD with and without agoraphobia symptom severity (post-treatment: *P*>.99, follow-up: *P*=.84). In the exposure app group, 35% (n=11) of participants showed reliable improvement in overall PD with and without agoraphobia symptom severity at post-treatment (meditation app: n=2, 6%; waiting list: n=2, 7%; *P*=.002*)*. The dropout rate after 5 weeks was 14% (5/37) in the exposure app group (meditation app: 3/37, 8%; waiting list: 6/37, 16%; *P*=.68). No adverse outcomes were reported in the exposure app group.

**Conclusions:**

Our findings suggest that app-guided exposure therapy can be a useful further treatment option in addition to established psychological and pharmacological strategies. However, superiority compared to the disorder-unspecific meditation app remains unclear.

## Introduction

A panic disorder is characterized by the repeated occurrence of unexpected panic attacks, by worries about the recurrence or consequences of another panic attack, and by behavioral changes related to these worries [1]. Panic disorders often occur comorbidly with agoraphobia, which is defined by a strong fear and avoidance of situations in which escape is difficult or help is not immediately available in the event of panic-like symptoms, such as being in public or crowded spaces or out of the house alone [1]. According to the World Mental Health Surveys, the estimated cross-national lifetime prevalence of panic disorder is approximately 1.7% [2] and that of agoraphobia is 1.5% [3]. Both disorders have a high risk of becoming chronic if left untreated [1].

International guidelines recommend treating panic disorders (PDs) with and without agoraphobia with cognitive behavioral therapy (CBT) and pharmacotherapy [4-7]. Psychological models highlight the role of dysfunctional appraisal of panic symptoms as severely threatening and the presence of fear of fear in PD with and without agoraphobia [8]. Exposure therapy aims at correcting these maladaptive psychological patterns by systematically confronting patients with their panic symptoms and associated fears. Interoceptive exposure and in vivo exposure are considered core features of CBT for PD with and without agoraphobia, and a component network meta-analysis identified interoceptive exposure as a particularly effective component of such therapies [7,9].

A range of internet- or mobile-based interventions (IMIs) has been developed for PD with and without agoraphobia, and international guidelines advise using CBT-based IMIs either as a stand-alone treatment or as a companion intervention during psychotherapy [5-7]. The terms digital CBT or internet-based CBT are frequently used as alternative terminology for this type of IMIs [5,7]. IMIs are generally considered a useful and important addition to the spectrum of treatments available for PD with and without agoraphobia and other anxiety disorders. They can be used flexibly at all locations and times, offer the possibility of pseudonymous use, and might empower patients by increasing perceived control and self-efficacy. Further, they can be tailored to specific audiences (eg, by offering multiple languages) and, in particular, in the case of mobile interventions, could improve the transfer of therapeutic content into daily life, for example, by facilitating regular exercises [10,11]. The latter could be especially relevant for PD with and without agoraphobia, as patients often experience difficulties leaving their homes and exposure exercises are a key therapeutic component.

Two recent meta-analyses [12,13], with 16 and 13 included controlled studies, respectively, investigated the effectiveness of IMIs for individuals with PD with and without agoraphobia. These meta-analyses identified only 2 randomized controlled trials (RCTs) that investigated mobile interventions, and none of these studies focused specifically on exposure therapy [14,15]. Both meta-analyses found that compared to passive control groups, such as waiting list (WL), IMIs were highly effective in reducing primary panic and agoraphobia symptoms. Further, medium to large effect sizes in favor of IMIs were also found for secondary outcomes such as reduction in depression and anxiety symptoms and increase in quality of life (QoL). However, none of the trials comparing IMIs and passive control groups included a follow-up assessment. When comparing IMIs to other active treatment groups, mainly face-to-face individual or group CBT, no significant differences were found in panic and agoraphobia symptoms, comorbid depression, anxiety, or QoL at post-assessment or follow-up. Overall, however, the number of eligible trials included in both meta-analyses is relatively small, and existing trials show considerable differences in terms of type, duration, and amount of therapist guidance of the interventions compared. More research, especially the conduction of further high-quality, rigorously conducted RCTs with appropriate control interventions and follow-up assessments, is therefore needed; in particular, this applies to studies featuring mobile interventions.

The objective of our longitudinal RCT was to evaluate the efficacy of a mobile app with a particular emphasis on interoceptive and in-vivo exposure for individuals with PD with and without agoraphobia. We compared a group of patients using the exposure therapy app with a passive (WL) and an active control group using a nonspecific mobile app for mindfulness meditation. Changes in panic and agoraphobia symptoms were the primary outcome parameters. Secondary outcome parameters included symptoms of depression, anxiety, stress, QoL, and self-efficacy expectations. We hypothesized that using the exposure therapy app would result in a significant symptom reduction, particularly in comparison to the WL group, in which we generally did not expect symptom improvements. Furthermore, we anticipated that the exposure therapy app would lead to greater symptom reduction than the meditation app, since the latter is not an intervention specifically designed for the treatment of PD with and without agoraphobia. Our study extends the current evidence in several important ways: First, it evaluates a purely mobile IMI, a type of intervention that is still underrepresented in recent meta-analyses [12,13]. Second, by evaluating 2 control conditions – a passive WL and an active, but not disorder-specific intervention – the study might help to disentangle general IMI effects from specific effects of mobile-delivered exposure therapy in PD with and without agoraphobia. And third, in contrast to previous IMI studies that typically studied broad CBT interventions featuring a wider range of therapeutic interventions from cognitive restructuring to stress management, the current RCT specifically focused on facilitating exposure, a key component of disorder-specific CBT in PD with and without agoraphobia.

## Methods

### Design

In this RCT, participants were randomly allocated to 3 equally sized groups: a group that received app-based exposure therapy, a group that received app-based mindfulness meditation (active control), and a WL group (passive control). Panic and agoraphobia symptoms, depression and anxiety, QoL, anxiety sensitivity, and self-efficacy expectations were assessed before the intervention (T_0_), after the 5-week intervention phase (T_1_), and at follow-up 13 weeks after T_0_ (T_2_).

### Ethical Considerations

The study was conducted in accordance with the Helsinki Declaration, approved by the University of Bonn’s medical ethics committee (protocol number: 321/20), and preregistered in the German Clinical Trials Register (DRKS00022204). Before the start of data collection, we decided to raise the upper age limit for participants from 50 years to 60 years and removed the Beck Depression Inventory from the list of outcomes to reduce survey length, as depressive symptoms are also addressed by the Depression, Anxiety and Stress Scale (DASS; refer to “Enrollment and Assessment” below for details). The study protocol was updated accordingly. All participants gave their written informed consent and could opt out of the study at any time. Study data were deidentified using participant codes for collecting interview and questionnaire data, as well as for app access. Participants did not receive any financial compensation for taking part in the study.

### Participants and Procedure

Individuals between the ages of 18 and 60 years who met the diagnostic criteria for a PD with and without agoraphobia were eligible to participate in the study. Current psychotherapeutic treatment (face-to-face individual or group therapy) was an exclusion criterion. Other exclusion criteria were acute psychosis, pregnancy, unstable cardiovascular disease, inadequately controlled hypertension, other medical conditions that precluded the administration of exposure therapy, and insufficient knowledge of the German language, as we evaluated German versions of the studied apps. Mobile device literacy was not explicitly specified as an inclusion criterion but implicitly required for study participation. Participants were allowed to continue psychiatric medication during the study.

Recruitment and data collection for the study took place between November 2020 and January 2023 (first participant in: November 17, 2020 and last participant out: January 11, 2023). Participants were recruited via the outpatient clinic of the Department of Psychiatry and Psychotherapy of the University Hospital Bonn; by advertising among psychotherapists, psychiatrists, general practitioners, self-help groups, psychological study counseling; and via social media and newspapers. Since the study was conducted entirely online, the participants were recruited nationwide in Germany as well as in Austria and Switzerland.

A total of 210 individuals expressed interest in the study and were contacted by the study team. The study team outlined the study design and presented the inclusion and exclusion criteria. After this initial step, 117 individuals progressed further and underwent the full eligibility assessment. To establish the diagnosis of PD with and without agoraphobia and to determine comorbid disorders, participants underwent a structured clinical interview at the baseline assessment (T_0_). The clinical interview was based on the Brief Diagnostic Interview for Mental Disorders Mini-DIPS [16] and carried out remotely, using a secure video communication platform [17]. If the diagnostic criteria were not met or any exclusion criterion was fulfilled, study participation was terminated at this point. If participants met the diagnostic criteria for PD with and without agoraphobia and did not show any exclusion criterion, we randomly assigned them to one of the 3 groups by drawing from an urn. The study team prepared the urn prior to the start of the study, and the allocation sequence was unpredictable. After screening, 111 individuals were included and assigned to 1 of the 3 groups; recruitment was stopped after reaching this prespecified sample size. Following the baseline assessment, the 5-week intervention period started for all included participants. During the intervention period, participants received weekly invitations to short surveys to assess usage patterns via SMS. At the end of the intervention period, all participants received a link to a post-intervention survey via email (T_1_). At T_2_, all participants received a link to a follow-up survey. Once the participants had completed this questionnaire, participation in the study was completed. Participants in the WL group equally underwent assessments at T_0_, T_1_, and T_2_ and could request access to one of the 2 apps following T_2_.

### Enrollment and Assessment

In total, 6 members of the study team rated the Mini-DIPS interview and performed randomization (1 physician, 1 postgraduate psychologist, 4 advanced-level psychology or medical students under supervision). Raters received training and conducted mock interviews before the first rating. All self-report data was obtained via an online survey tool [18].

### Primary Study Outcomes

Primary outcomes were panic and agoraphobia symptoms based on the following self-reported questionnaires:

#### Panic and Agoraphobia Scale

The Panic and Agoraphobia Scale (PAS) is a 13-item scale measuring overall symptom severity in individuals diagnosed with PD with and without agoraphobia [8]. The 13 items cover the frequency and severity of panic attacks, agoraphobic avoidance, anticipatory anxiety, functional impairment, and worry related to somatic health. The PAS is recommended by guidelines [5,19] and has been widely used in clinical trials (eg, [20-22]). The total sum score ranges from 0 to 52, with scores above 8 indicating mild, above 18 intermediate, above 28 severe, and above 39 very severe symptoms.

#### Agoraphobic Cognitions Questionnaire and Body Sensations Questionnaire

The Agoraphobic Cognitions Questionnaire (ACQ) and the Body Sensations Questionnaire (BSQ) are companion measures focusing on the concept of fear of fear in PD with and without agoraphobia [23-25]. The ACQ thereby addresses negative or catastrophic thoughts associated with fear and nervousness, including the expectation of severe somatic illness such as stroke or brain tumor, and the expectation of loss of control. The ACQ is provided as a 14-item scale and its score ranges from 1 to 5. The BSQ, in turn, assesses anxiety associated with bodily sensations typically present during panic attacks (eg, palpitations, dizziness, or depersonalization) on a 17-item scale and its score ranges from 1 to 5.

#### Mobility Inventory

The Mobility Inventory (MI) [25-27] outlines 27 situations often avoided by individuals with PD with and without agoraphobia like supermarkets, buses, or crowded places. Participants rate their degree of avoidance for each of these situations separately for avoidance alone and avoidance when accompanied by a trusted person. The derived scores range from 1 to 5.

#### Texas Safety Maneuver Scale

The Texas Safety Maneuver Scale (TSMS) [28-30] measures dysfunctional strategies used to reduce anxiety and panic in situations perceived as dangerous (eg, checking the location of nearby hospitals or distracting oneself through music). The TSMS lists 50 common safety maneuvers and prompts participants to rate the degree to which they use these strategies. The possible overall score ranges between 0 and 250.

### Secondary Study Outcomes

Secondary outcomes were general anxiety, depression, and stress symptoms, QoL, anxiety sensitivity, and self-efficacy expectations, and assessed by the following self-reported questionnaires:

#### World Health Organization Quality of Life

The 26-item BREF version of the World Health Organization Quality of Life (WHO-QOL) scale is a self-report questionnaire developed by the World Health Organization [31,32]. It assesses the global health status of patients across 4 health domains: physical health, psychological QoL, social relationships, and environmental QoL. The total scores for each domain range from 0 to 100.

#### DASS-21

The DASS-21 [33,34] consists of 21 items, equally divided into 3 subscales: depression, anxiety, and stress. The subscales each range from 0 to 42 (sum score multiplied by 2 to retain consistency with scoring of the long version of DASS).

#### Anxiety Sensitivity Index-3

The Anxiety Sensitivity Index-3 (ASI-3) [35-37] measures anxiety sensitivity, a construct similar to fear of fear. The questionnaire contains 18 items, and its overall scale varies between 0 and 72.

#### General Self-Efficacy Scale

The General Self-Efficacy Scale (GSES) [38-40] was used to assess perceived general self-efficacy, that is, the extent to which people believe they are able to successfully cope with challenging life situations. The GSES is administered as a 10-item questionnaire, and its total sum score ranges from 10 to 40.

### Interventions

Participants assigned to the exposure app group and to the meditation app group each were instructed to use the respective mobile app for a period of 5 weeks. During this period, they received no further instructions from the study team regarding the app content besides a short introduction at baseline (ie, self-guided therapy). To minimize the risk of performance bias, none of the 2 apps was presented as superior to the participants during the onboarding procedure. However, we could not guarantee that participants were kept completely blind, as information documents about the study stating that the exposure therapy intervention was the intervention of interest had to be made available online (eg, in the study registration) and had to be disclosed to obtain informed consent. Both apps were available for Android and iOS devices. After the 5-week intervention period, access to the app was not restricted, but no further instructions were given, so that participants could continue using their respective app as they wished.

### Exposure Therapy Intervention

**Figure 1.**
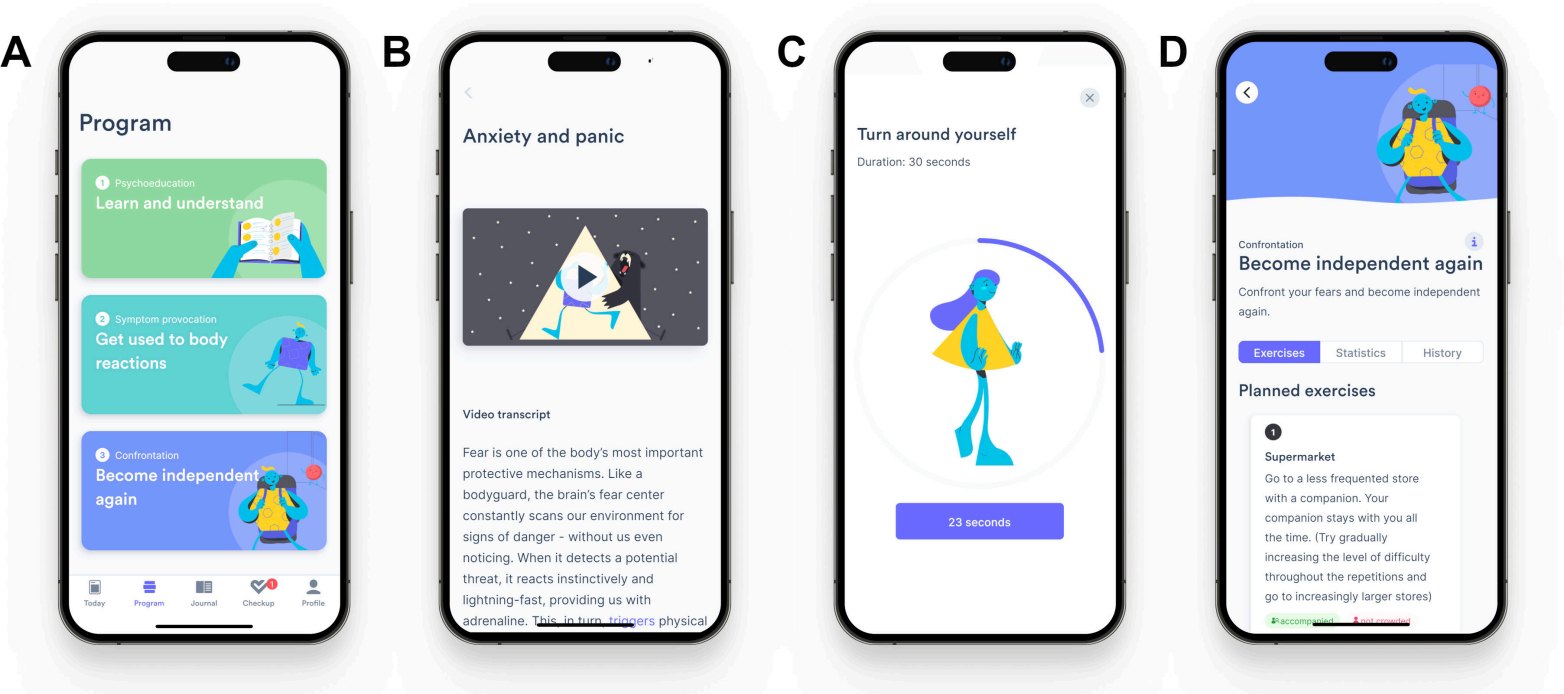
Visualization of the exposure therapy app. Visualization copyright: Mindable Health GmbH.

Participants in the exposure app group received free access to Mindable: Panic & Agoraphobia [41], an e-health application otherwise available to individuals with PD with and without agoraphobia in Germany on prescription [42]. The app is based on the manual for cognitive-behavioral exposure therapy published by Lang et al [30] and focuses on preparing, accompanying, and debriefing various exposure exercises. After working through a base module featuring psychoeducative content (eg, psychological models of causes, maintaining factors, and consequences of the disorder), patients are guided through a second module focusing on interoceptive exposure (eg, accustoming to self-provoked body sensations such as dizziness or tachycardia) and a third module facilitating in-vivo exposure of agoraphobic situations. For the third module, patients define situations relevant for themselves (eg, public transportation, supermarket, and elevators) and are encouraged to gradually increase exposure difficulty (eg, first accompanied and later alone). Content is delivered through videos and accompanying texts. Patients interact with the application, for example, by answering self-reflection questions, logging anxiety during exposure exercises, and repetitively filling short symptom questionnaires. Patients are instructed to use the app daily and schedule at least one exposure exercise per week. Customizable mobile-operating-system-based notifications reminded participants to frequently use the app and complete scheduled tasks. We recommended participants to work through the 3 therapy modules chronologically, starting with the psychoeducation module in the first week. A study-specific “frozen” version of the app was used and not changed throughout the study. Figure 1 presents exemplary screenshots of the exposure therapy app. Panel A shows an app overview featuring the 3 main modules: psychoeducation, interoceptive exposure, and in-vivo exposure. Panel B features an exemplary video transcription from the psychoeducation module, and panel C an exemplary interoceptive exposure exercise focused on the elicitation of dizziness. An exemplary in-vivo exposure exercise guiding a visit to a supermarket together with a trusted person is displayed in panel D. The operating language was set to German for the study.

### Mindfulness Meditation Intervention

Participants in the meditation app group received free access to 7Mind [43], a meditation app which is otherwise available in Germany through selected health insurances, some employers, and on a self-payment basis. The application teaches mindfulness meditation through a range of courses (eg, “foundation” and “from stress to silence”) and themed exercises (eg, “falling asleep” and “bus or train”). The app dynamically recommends content and provides advice. Participants can schedule meditations within the program and receive reminders. The intervention is not specifically focused on PD with and without agoraphobia. Participants were encouraged to use the app daily and to choose courses and relaxation exercises according to their own needs and interests.

### Statistical Analyses

To evaluate the results under real-world conditions, we conducted an intention-to-treat (ITT) analysis for all primary and secondary parameters, in which we included all available data from all randomized participants. In addition, to examine the results under ideal conditions, we conducted an additional completer analysis on all primary outcome parameters. In both analyses, separate linear mixed effects models (LMM) were calculated for each analyzed outcome parameter. The model specification controlled for baseline values by including the factor time (T_0,_ T_1_, and T_2_) and the interaction of group (exposure, meditation, and WL) and time [44]. Further, participant ID was added as a random effects term, and age, gender, and education served as covariates. Post hoc tests were conducted by running Bonferroni-corrected *t* tests on the estimated marginal means from the model. All reported statistical tests are based on a significance level of ɑ=.05. Partial Eta-squared (η_p_^2^) was used as a measure of LMM effect sizes. For significant effects, post hoc *t* test effect sizes were reported as Cohen *d*, using the estimated marginal means and the pooled SD from the observed means. We analyzed reliable changes based on the reliable change index suggested by Jacobson and Truax [45] using normative data for the PAS [8]. CIs for the number needed to treat (NNT) were estimated using the Wald method [46]. All statistical analyses were conducted in R 4.2.2 [47]. The packages lme4 [48] and emmeans [49] were used for model estimation and post hoc testing.

### Missing Data Handling and Exclusions

For the ITT analysis, data of all 37 participants per group were analyzed, including those with missing data at 1 or 2 timepoints. A detailed summary of the number of complete cases that were included in the ITT analysis for each questionnaire per group and timepoint can be found in the supplementary data S1 in Multimedia Appendix 1.

For the completer analysis, we considered only those participants who had no missing data in any questionnaires at all 3 time points. In total, 7 participants (3 in exposure app group, 2 in meditation app group, and 2 in WL group) indicated at the T_1_ and T_2_ assessment that, in addition to the apps evaluated in the study, they had used other meditation or therapy apps to reduce their PD with and without agoraphobia symptoms. We also excluded these individuals from the completer analysis. Thus, the completer analysis included 14 participants from the exposure app group, 25 participants from the meditation app group, and 27 participants from the WL group.

## Results

### Baseline Characteristics

Demographic information, diagnostic status, and baseline symptom scores for all 3 groups are summarized in Table 1. A total of 111 participants (mean age: 36.78, SD 12.58; n=81, 73% female) were randomized. Of these 111 participants, 59% (n=65) fulfilled criteria for combined PD with and without agoraphobia, 22% (n=24) from mere panic disorder and 20% (n=22) from mere agoraphobia. Compared to the WL group, significantly more participants (*P*=.03) in the meditation app group had concomitant other anxiety disorders (eg, social anxiety disorder and generalized anxiety disorder). All other demographic and diagnostic status variables showed no significant group differences. The mean PAS score across all groups was 22.13 (SD 9.06) at baseline, corresponding to intermediate symptom severity. A significant baseline group difference was detected for the PAS score (*P=*.02), driven by significantly lower scores in the meditation app group compared to the WL group (exposure group vs WL group: *P=.*84; meditation group vs WL group: *P=.*02; mediation group vs exposure group: *P=.*07). Of note, however, we controlled for baseline differences in all statistical models (refer to the “Statistical Analyses” section above for details). There were no other significant baseline group differences in any of the other primary or secondary outcomes.

**Table 1.** Demographic and clinical characterization.

Demographics	App-based exposure therapy (n=37)	App-based mindfulness meditation (n=37)	Waiting list (n=37)	*P*^a^ values
Age (years)				.06
Mean (SD)	39.05 (13.28)	38.49 (12.14)	32.81 (11.63)	
Range	20‐60	19‐59	19‐57	
Gender, n (%)				.44
Male	7 (19)	9 (24)	12 (32)	
Female	29 (78)	27 (73)	25 (68)	
Not specified	1 (3)	1 (3)	0 (0)	
Education, n (%)				.78
Primary or below	0 (0)	0 (0)	0 (0)	
Lower secondary education	13 (35)	7 (19)	6 (16)	
Higher secondary education	12 (32)	19 (51)	16 (43)	
University degree	11 (30)	10 (27)	14 (38)	
Postgraduate	1 (3)	1 (3)	1 (3)	
PD^b^ with and without agoraphobia diagnostic status, n (%)				.32
PD and agoraphobia	25 (68)	17 (46)	23 (62)	
PD only	7 (19)	9 (24)	8 (22)	
Agoraphobia only	5 (14)	11 (30)	6 (16)	
Comorbidities, n (%)				
Other concomitant anxiety disorder	25 (68)	29 (78)	19 (51)	.048^c^
Concomitant affective disorder	6 (16)	1 (3)	6 (16)	.43
Other concomitant psychiatric diagnosis	18 (49)	12 (32)	16 (43)	.54
PD with and without agoraphobia symptom severity, mean (SD)				
PAS^d^	23.3 (7.19)	18.7 (9.89)	24.44 (9.09)	.02^e^
ACQ^f^	2.29 (0.60)	2.14 (0.68)	2.11 (0.53)	.41
BSQ^g^	2.89 (0.62)	2.69 (0.84)	2.94 (0.71)	.30
MI^h^ accompanied	2.09 (0.80)	1.96 (0.87)	1.78 (0.66)	.22
MI alone	2.63 (0.94)	2.45 (1.11)	2.29 (0.84)	.32
TSMS^i^	117.97 (42.91)	98.62 (42.47)	105.84 (41.19)	.14
Secondary outcomes, mean (SD)				
WHO-QOL^j^ psychological	54.84 (15.45)	58.22 (12.36)	55.41 (16.28)	.58
WHO-QOL physical	66.31 (17.22)	66.80 (15.27)	65.44 (17.82)	.94
WHO-QOL social	62.84 (20.93)	65.77 (20.39)	64.41 (17.96)	.82
WHO-QOL environmental	73.40 (12.25)	71.03 (12.73)	74.41 (12.21)	.49
DASS^k^ depression	15.08 (10.88)	11.14 (9.56)	15.30 (10.08)	.15
DASS stress	21.78 (10.02)	17.62 (9.07)	20.38 (10.25)	.18
DASS anxiety	20.97 (10.54)	17.57 (9.49)	21.89 (8.88)	.13
ASI^l^	40.19 (13.37)	36.68 (12.86)	35.86 (12.88)	.32
GSES^m^	25.35 (4.64)	25.95 (3.95)	26.54 (5.13)	.54

a *P*-column states ANOVA (continuous variables) or* χ*2 (categorical measures) test results. If *P*<.05, we conducted post hoc tests (pairwise comparisons).

b PD: panic disorder.

c Significant post hoc test: meditation versus waiting list (*P*=.03), other post hoc test not significant.

d PAS: Panic and Agoraphobia Scale.

e Significant post hoc test: meditation vs waiting list (*P*=.02), other post hoc test not significant, statistical models controlled for baseline differences.

f ACQ: Agoraphobic Cognitions Questionnaire.

g BSQ: Body Sensations Questionnaire.

h MI: Mobility Inventory.

i TSMS: Texas Safety Maneuver Scale.

j WHO-QOL: World Health Organization Quality of Life.

k DASS: Depression, Anxiety and Stress Scale.

l ASI: Anxiety Sensitivity Index.

m GSES: General Self-Efficacy Scale.

### Response and Usage Patterns

**Figure 2.**
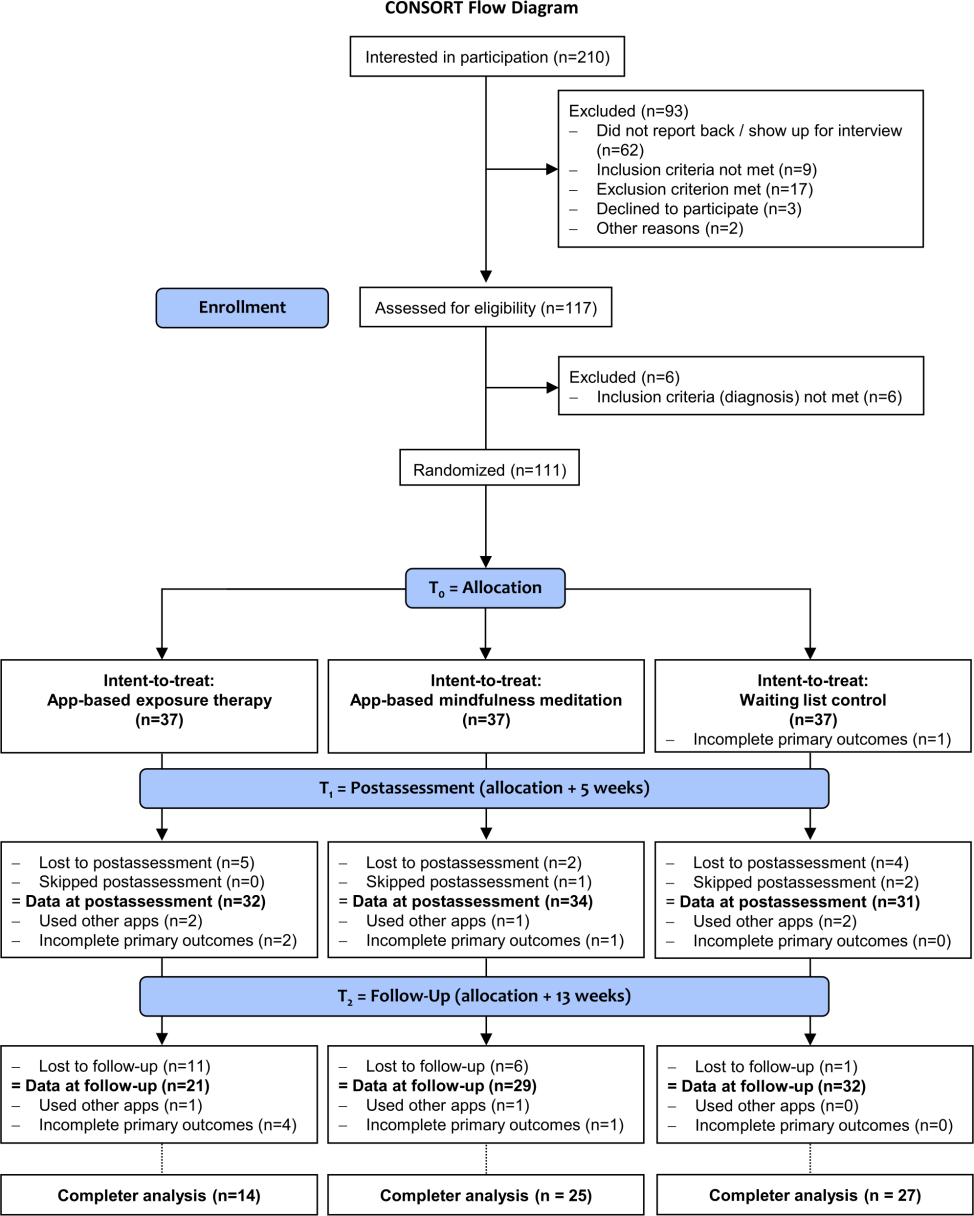
Consolidated Standards of Reporting Trials (CONSORT) flow diagram.

From a total of 111 participants enrolled (37 participants per group), 11 participants dropped out of the study at T_1_. Furthermore, 3 participants did not participate at the T_1_ assessment but returned for T_2_. In total, T_1_ data was missing for 5 (14%) participants in the exposure app group, 3 (8%) participants in the meditation app group, and 6 (16%) participants in the waiting list group. At T_2_, an additional 18 participants had dropped out (total dropout rate in the exposure app group at T_2_: n=16, 43%; meditation app group: n=8, 22%; waiting list group: n=5, 14%). In addition, at all 3 time points, individual participants only partially completed the questionnaires. Dropout rates at T_1_ did not significantly differ between groups (Fisher exact test: *P*=.68). At T_2_, we found significant differences in dropout rates between the groups (Fisher exact test: *P*=.01). Both at T_1_ and at T_2_, participants who dropped out did not significantly differ on any of the measured baseline characteristics including symptom severity (refer to Table 1 for a list of the tested parameters). For detailed information on the composition of dropouts, refer to the Consolidated Standards of Reporting Trials (CONSORT) flow diagram (Figure 2).

On a weekly basis, we evaluated usage patterns by asking participants how often and how long they used the app. The most selected category for frequency (average across all 5 wks) was “Several times a week” for both the exposure group (42.3%) and the meditation group (41.2%). Participants selected “daily” more often in the meditation app group than in the exposure app group (29.2% vs 12.0%). Notably, members of the exposure app group selected “Never” more frequently than those in the meditation app group (19.1% vs 8.7%).

The average duration per use was similar in both groups. Here, the most selected category was “less than 15 minutes” in both the exposure group (50.0%) and the meditation group (50.2%), followed by “15 to 30 minutes” (34.8% and 36.6%, respectively). On average, only 1.82% of participants in the exposure group and 0% in the meditation group used the respective app for “more than 1 hour” per use. For detailed information on usage patterns per week, refer to Table S2 in Multimedia Appendix 2.

### Primary Outcomes

**Figure 3.**
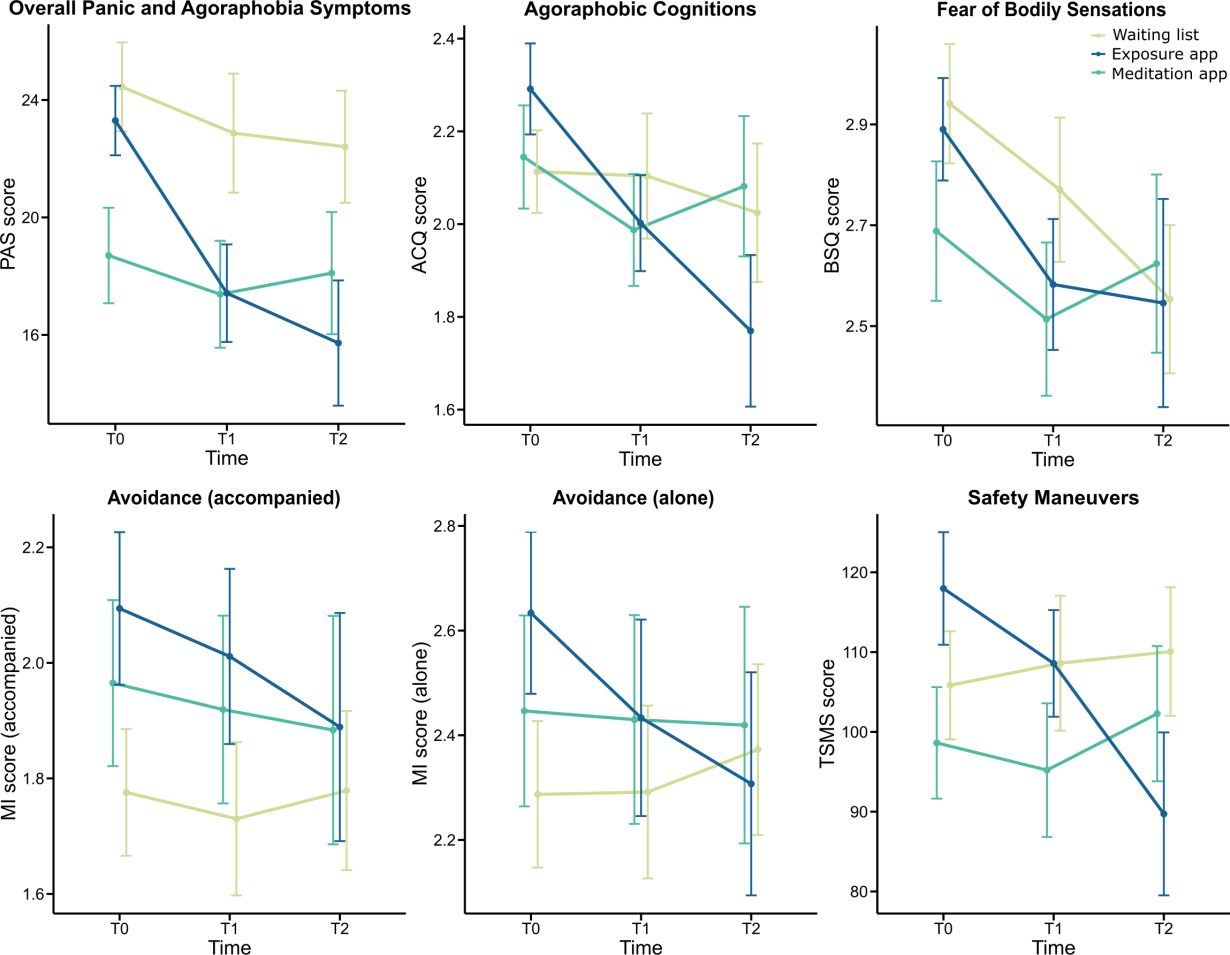
Changes in primary outcome means in each group across the 3 time points. Error bars represent the standard error of the mean. ACQ: Agoraphobic Cognitions Questionnaire; BSQ: Body Sensations Questionnaire; MI: Mobility Inventory; PAS: Panic and Agoraphobia Scale; TSMS: Texas Safety Maneuver Scale.

Changes in PD with and without agoraphobia symptom severity during the study are depicted in Figure 3. In the exposure app group, scores decreased from T_0_ to T_1_ as well as from T_1_ to T_2_ on all 6 symptom severity scales. Also, the meditation app group showed a decrease in all 6 symptom scores from T_0_ to T_1_; however, here the decreases were descriptively smaller, and they increased again at T_2_ in 4 of the measures (overall PD with and without agoraphobia symptoms, agoraphobic cognitions, fear of bodily sensations, and safety maneuvers). The WL group showed varying courses of outcome with no clear overall tendencies.

Table 2 summarizes the LMM results of our ITT analysis (for more detailed results, refer to supplementary data in Multimedia Appendix 3). For overall PD with and without agoraphobia symptoms (PAS), we found a significant time×group interaction (*F*_6,148.48_=3.00, *P*=.009, η_p_^2^=0.11). Post hoc *t* tests revealed significant differences between the exposure app group and the WL group at both T_1_ (*t*_177_=2.48, *P*=.04, *d*=0.55) and T_2_ (*t*_211_=2.50, *P*=.04, *d*=0.60), but no other significant differences in group comparisons. In addition, for the exposure app group, we observed significant reductions in overall PD with and without agoraphobia symptoms between T_0_ and T_1_ (*t*_174_=4.24, *P*<.001, *d*=0.72) and between T_0_ and T_2_ (*t*_181_=4.18, *P*<.001, *d*=0.90), while for all other within-group comparisons, we did not find any significant differences.

**Table 2.** Estimated mean differences of primary outcomes–intention-to-treat analyses.

	T_1_ (allocation+5 wk)				T_2_ (allocation+13 wk)			
Comparison^a^	Δ^b^ (SE)	95% CI	*P* values	Cohen *d*	Δ^b^ (SE)	95% CI	*P* values	Cohen *d*
Overall panic and agoraphobia symptoms								
Exposure app–Waiting list	−5.64 (2.27)	−11.13 to −0.14	.04	0.55	−6.15 (2.46)	−12.10 to −0.21	.04	0.60
Exposure app–Meditation app	−1.16 (2.21)	−6.50 to 4.19	>.99	0.12	−2.65 (2.45)	−8.56 to 3.26	.84	0.25
Waiting list–Meditation app	4.48 (2.23)	−0.91 to 9.87	.14	−0.41	3.50 (2.26)	−1.95 to 8.96	.37	−0.32
Safety maneuvers								
Exposure app–Waiting list	0.74 (9.98)	−23.44 to 24.93	>.99	−0.02	−15.90 (10.48)	−41.24 to 9.45	.39	0.35
Exposure app–Meditation app	4.79 (9.72)	−18.76 to 28.35	>.99	−0.11	−12.81 (10.36)	−37.85 to 12.23	.65	0.29
Waiting list–Meditation app	−4.05 (9.82)	−19.73 to 27.84	>.99	−0.08	3.90 (9.89)	−20.86 to 27.04	>.99	−0.07

a All models control for baseline differences.

b *Δ*=Group differences based on estimated marginal means.

For safety maneuvers (TSMS), we likewise found a significant time×group interaction (*F*_6,142.69_=2.88, *P*=.01, η_p_^2^=0.11). Here, our post hoc *t* tests did not reveal any significant between-group effects, but significant within-group decreases for the exposure app group between T_0_ and T_1_ (*t*_172_=2.74, *P*=.02, *d*=0.32) and T_0_ and T_2_ (*t*_175_=4.69, *P*<.001, *d*=0.62). All other within-group comparisons revealed no significant differences.

Regarding agoraphobic cognitions, fear of bodily sensations, and avoidance, we found no significant time×group interactions in the ITT analysis. However, across groups, the LMM for fear of body sensations (BSQ) revealed a significant main effect of time (*F*_2,173.61_=5.06, *P*=.007, η_p_^2^=0.06).

In the additional completer analysis, we again found a significant time×group interaction (*F*_6,91.90_=2.69, *P*=.02, η_p_^2^=0.15) for overall PD with and without agoraphobia symptom severity (PAS). When following up the interaction with post hoc *t* tests, we, however, did not find significant between-group differences at any of the 3 time points. Regarding within-group comparisons, we found a significant reduction in overall PD with and without agoraphobia symptom severity in the exposure app group between T_0_ and T_1_ (*t*_126_=3.50, *P*=.002, *d*=0.79) and between T_0_ and T_2_ (*t*_126_=3.65, *P*=.001, *d*=0.84), but no other within-group comparison reached level of significance. Regarding safety maneuvers (TSMS), we again observed a significant time×group interaction (*F*_6,91.90_=2.94, *P*=.01, η_p_^2^=0.16) that we followed up using post hoc *t* tests. These indicated a significant within-group decrease for the exposure app group between T_0_ and T_2_ (*t*_126_=3.95, *P*<.001, *d*=0.56), while all other post hoc tests did not show significant effects. Detailed results of the completer analysis can be found in supplementary data in Multimedia Appendix 4.

### Secondary Outcomes

**Figure 4.**
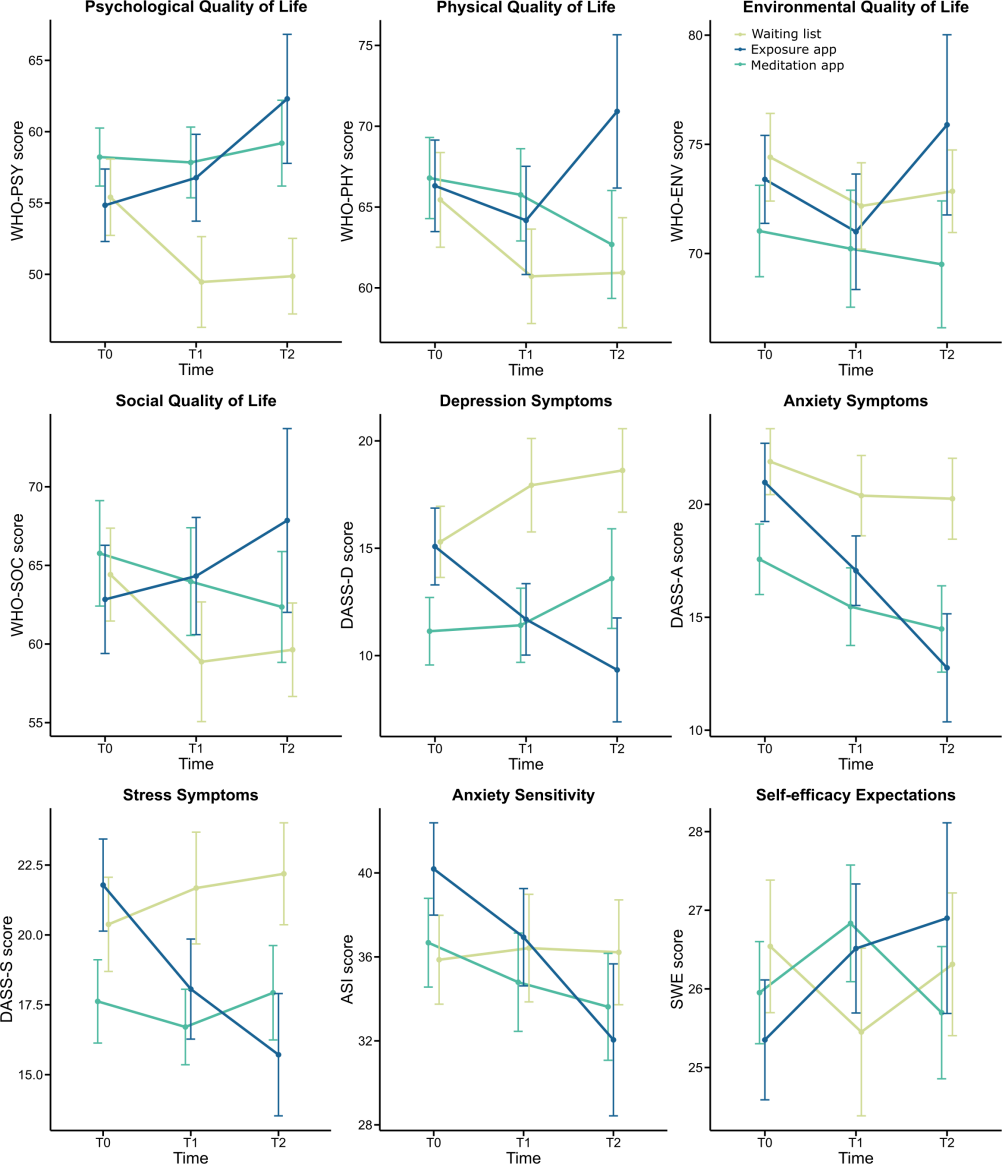
Changes in secondary outcome means in each group across the 3 time points. Error bars represent the SE of the mean. ASI: Anxiety Sensitivity Index; DASS-D: Depression, Anxiety and Stress Scale – Anxiety; DASS-D: Depression, Anxiety and Stress Scale – Depression; DASS-S: Depression, Anxiety and Stress Scale – Stress; WHO-ENV: World Health Organization Quality of Life – Environmental subscale; WHO-PSY: World Health Organization Quality of Life – Psychological subscale; WHO-PHY: World Health Organization Quality of Life – Physical subscale; WHO-SOC: World Health Organization Quality of Life – Social subscale.

Changes in secondary outcomes are depicted in Figure 4. Descriptively, the exposure app group improved in all secondary outcomes over the course of the study, while most measures stayed stable or even deteriorated in the other 2 groups.

In the ITT analysis, the LMM on psychological QoL (as measured by the psychological subscale of the WHO-QOL) revealed a significant main effect of time (*F*_2,175.68_=4.45, *P*=.01, η_p_^2^=0.05) as well as a significant time×group interaction (*F*_6,144.29_=3.12, *P*=.007, η_p_^2^=0.11). The post hoc *t* tests attributed this interaction to a significantly higher psychological QoL in the exposure app group than in the WL group at T_2_ (*t*_160_=–2.88, *P*=.01, *d*=0.63), since all other between-groups comparisons showed no significant differences. Moreover, for the exposure app group, we found significant within-group increases in the psychological QoL between T_0_ and T_2_ (*t*_178_=−3.14, *P*=.006, *d*=0.36) and between T_1_ and T_2_ (*t*_175_=−2.98, *P*=.01, *d*=0.32). For the WL group, in turn, we found significant decreases in psychological QoL between T_0_ and T_1_ (*t*_175_=2.54, *P*=.04, *d*=−0.26) and between T_0_ and T_2_ (*t*_175_=2.59, *P*=.03, *d*=−0.28), while all other within-group comparisons showed no significant differences.

Further, there was a significant time×group interaction for depressive symptoms as measured by the DASS questionnaire (*F*_6,148.84_=3.00, *P*=.009, η_p_^2^=0.11). Our post hoc between-group comparison thereby revealed significantly lower depressive symptoms in the exposure app group compared to the WL group at T_2_ (*t*_206_=3.09, *P*=.007, *d*=0.75). Moreover, within the exposure app group, we found a significant decrease in the severity of depressive symptoms from T_0_ to T_2_ (*t*_184_=2.87, *P*=.01, *d*=0.48), while all of our other comparisons did not show any significant effects.

Regarding our ITT analyses on all remaining secondary outcomes (physical, social, and environmental QoL, stress and overall anxiety, anxiety sensitivity, and perceived general self-efficiency), we found no significant time effects or time×group interactions. Detailed results of our analyses for secondary outcomes can be found in Multimedia Appendix 5.

### Response and Safety Analyses

Because we found a significant between-group effect for overall PD with and without agoraphobia symptoms, we analyzed individual change for each participant based on this outcome (refer to Tables3  4). At T_1_, we observed reliable improvement for 35% (n=11) of participants in the exposure app group (meditation app: n=2, 6%; WL: n=2, 7%; Fisher exact test: *P*=.002). In the exposure app group, 23% (n=7) of participants showed reliable improvement to a score below the PAS cut-off value of 8 suggested for remission [8] compared to 3% (n=1) in the WL group and no participant in the meditation app group (Fisher exact test: *P=*.12). Compared to WL, these differences translate into a NNT of 3.47 for reliable improvement and 5.20 for reliable improvement to remission (compared to meditation app: NNTs of 3.39 and 4.43, respectively). Follow-up data showed a similar pattern with an NNT of 3.85 for reliable improvement compared to WL and an NNT of 3.16 compared to the meditation app. However, 3 remitted participants in the exposure app group dropped out between T_1_ and T_2_, and we observed lower rates of remission at T_2_ compared to T_1_ for the exposure group. NNT CIs for this measure included ± ∞, implying that no significant differences in remission rates were observed between the groups at T_2_.

**Table 3.** Response analyses: overall panic and agoraphobia symptoms.

	Post-assessment (allocation+5 wk)	Follow-up (allocation+13 wk)
Reliable improvement^a^		
Exposure app	11 of 31 (35%)	7 of 18 (39%)
Meditation app	2 of 34 (6%)	2 of 29 (7%)
Waiting list	2 of 30 (7%)	4 of 31 (13%)
Reliable improvement to remission^a^		
Exposure app	7 of 31 (23%)	3 of 18 (17%)
Meditation app	0 of 34 (0%)	1 of 29 (3%)
Waiting list	1 of 30 (3%)	2 of 31 (6%)
Reliable deterioration^a^		
Exposure app	1 of 31 (3%)	0 of 18 (0%)
Meditation app	1 of 34 (3%)	3 of 29 (10%)
Waiting list	0 of 30 (0%)	2 of 31 (6%)

a Reliable change calculated based on the reliable change index suggested by Jacobson and Truax [45] using normative data for the Panic and Agoraphobia Scale [8] for all participants with sufficient data.

**Table 4.** Response analyses: NNT^a^ for overall panic and agoraphobia symptoms.

	Post-assessment (allocation+5 wk), NNT (95% CI)	Follow-up (allocation+13 wk), NNT (95% CI)
Reliable improvement^b^		
Exposure app–waiting list	3.47 (2.09 to 10.25)	3.85 (2.09 to 10.25)
Exposure app–meditation app	3.39 (2.07 to 9.10)	3.16 (1.78 to 13.06)
Waiting list–meditation app	127.50 (–∞ to –8.98) ∪ (7.87 to ∞)	16.65 (–∞ to –11.15) ∪ (4.77 to ∞)
Reliable improvement to remission^b^		
Exposure app–waiting list	5.20 (2.83 to 31.37)	9.79 (–∞ to –11.05) ∪ (3.39 to ∞)
Exposure app–meditation app	4.43 (2.68 to 12.72)	7.57 (–∞ to –19.10) ∪ (3.16 to ∞)
Waiting list–meditation app	30.00 (–∞ to –32.36) ∪ (10.25 to ∞)	33.30 (–∞ to –12.66) ∪ (7.19 to ∞)
Reliable deterioration^b^		
Exposure app–waiting list	31.00 (–∞ to –33.40) ∪ (10.59 to ∞)	–15.50 (–∞ to –6.62) ∪ (45.52 to ∞)
Exposure app–meditation app	351.33 (–∞ to –12.29) ∪ (11.48 to ∞)	–9.67 (–∞ to –4,67) ∪ (135.24 to ∞)
Waiting list–meditation app	–34.00 (–∞ to –11.60) ∪ (36.52 to ∞)	–25.69 (–∞ to –5.57) ∪ (9.84 to ∞)

a NNT=number needed to treat.

b Reliable change calculated based on the reliable change index suggested by Jacobson and Truax [45] using normative data for the Panic and Agoraphobia Scale [8] for all participants with sufficient data.

To assess the safety of the meditation app, we also analyzed data for reliable individual deterioration. At T_1_, 1 participant (3%) in the exposure app group showed reliable deterioration (meditation app: n=1, 3%; WL: n=0, 0%). The observed score for this participant increased from 12 (mild symptoms) to 24 (intermediate symptom severity). At T_2_, reliable deterioration was not observed in the exposure app group (meditation app: n=3, 10%; WL: n=2, 6%). Between-group NNT comparisons for reliable deterioration showed no significant group differences (all CIs included ± ∞). One participant from the WL group and 1 participant from the meditation app group were admitted to the hospital and subsequently dropped out of the study. No adverse events were reported to the study team in the exposure app group.

## Discussion

### Principal Findings

In this longitudinal RCT, we evaluated the efficacy of an exposure therapy app for PD with and without agoraphobia in comparison to an active (mindfulness meditation app) and passive (WL) control group. We found that the primary outcome parameter overall PD with and without agoraphobia symptom severity was significantly lower in the exposure app group compared to WL at both post-treatment and follow-up. Within the exposure app therapy group, the use of safety maneuvers declined between baseline and posttreatment and between baseline and follow-up. Regarding secondary outcomes, we found significant between-group effects for the exposure app versus waiting list comparisons in depressive symptoms and psychological QoL at follow-up.

### Primary Outcomes

The findings demonstrate the efficacy of the exposure app as a treatment for core symptoms of PD with and without agoraphobia. Between-group effect sizes compared to WL were medium according to the classification suggested by Cohen [50]. Treatment effects were sustained throughout the entire study period of 13 weeks. PD with and without agoraphobia symptom scores declined even further between post-assessment and follow-up, suggesting potential for further therapeutic gains in long-time users. Compared to WL, participants who used the exposure therapy app showed stronger improvement in overall severity of PD with and without agoraphobia symptoms. This finding is in line with a recently published RCT that investigated the same exposure therapy app and also found significantly greater improvement in overall PD with and without agoraphobia severity in the exposure app group compared to a waiting list control group [51].

Further, in the exposure app group, we also found a reduction in maladaptive safety maneuvers. These strategies have been found to contribute to the persistence of symptoms by reaffirming the assessment that the associated situations are dangerous and by preventing full exposure and subsequent anxiety reduction [28,52]. Safety maneuvers are explicitly addressed within the exposure app, and our findings suggest that participants engaged in less of these maneuvers following exposure app use. Descriptively, scores for the remaining symptom clusters associated with PD with and without agoraphobia (agoraphobic cognitions, fear of bodily sensations, and avoidance behavior) also declined within the exposure app group, but the effects failed to reach statistical significance. As the exposure therapy app possibly targets all these symptom subsets through framed interoceptive and in-vivo exposure, the overall improvement in PD with and without agoraphobia symptoms observed might have been the result of the sum of smaller improvements in all these symptom subsets. In consequence, the study might not have been sufficiently powered to detect significant group differences for the possibly smaller improvements in symptom subset scores.

In addition to comparing the app-based exposure therapy to WL, we evaluated its efficacy against an alternative active control intervention, the mindfulness meditation app. The mindfulness meditation app was not specifically designed to treat PD with and without agoraphobia but provides a similar experience of using a high-quality digital intervention. In theory, comparing the 2 apps should permit separating general effects of using digital interventions (eg, stemming from the sense of being able to self-treat symptoms with a novel type of intervention) from specific effects of app-guided exposure therapy. Descriptively, symptom scores declined more in the exposure app group compared to the mindfulness meditation app group. Additionally, significant within-group symptom reductions as well as significantly lower post-treatment symptoms compared to WL were only observed for the exposure therapy app. These observations seem to support the assumption that app-guided exposure therapy is specifically suitable as a digital intervention in PD with and without agoraphobia. However, direct comparisons between the exposure app and the meditation app did not demonstrate a statistically significant difference in symptom improvement favoring the exposure app group. One possible explanation might be that the meditation app itself had additional specific therapeutic effects exceeding those generally associated with app usage. These may have mitigated exposure therapy effects in between-group comparisons, which is supported by previous research that found positive effects of mindfulness meditation in anxiety disorders including PD with and without agoraphobia [53,54]. App usage pattern might have also played a role here. Our weekly survey indicated that more people in the meditation group than the exposure group used the app every day. This increased engagement could have had a reinforcing effect on the therapeutic benefit of the meditation app. Another possible explanation is that the degree to which exposure therapy effects exceed general effects associated with mobile intervention usage was too small to be detected with our study design and sample size. As previous literature on similar comparisons is very limited [12], it is difficult to judge what effect size is to be expected for this type of comparison, and our study might have been underpowered. Another aspect to consider is that recent meta-analyses showed that digital interventions in PD with and without agoraphobia are often provided as guided interventions [12]. Also, the exposure therapy app studied here was built based on material from a therapeutic manual intended to be used with therapist guidance [30]. The effects of using the intervention unguided might have been smaller than those of a guided intervention, and this might have resulted in insignificant comparisons with the meditation app. Furthermore, despite randomization, baseline PD with and without agoraphobia symptom severity scores were significantly lower in the meditation app group. Meditation might be particularly difficult to practice when symptoms are more severe. Therefore, meditation could have lower effects among participants with higher symptom scores, a subgroup that was underrepresented in the meditation app group of our study. In addition, our between-app comparisons suggested that advantages of the exposure app become more pronounced at follow-up compared to post-assessment. This suggests that our overall study period might have been too short to detect significant between-group effects of app-guided exposure therapy compared to the meditation app.

### Secondary Outcomes

Compared to WL, psychological QoL and depressive symptoms improved significantly among exposure therapy app participants at follow-up but not at post-treatment. The observed delayed significant improvement in these secondary outcomes might suggest that app-guided exposure therapy effects on these measures might be downstream effects of initial improvements in core PD with and without agoraphobia symptoms. The observed effects are partially consistent with the results of the 2 previous meta-analyses [12,13] which also found that IMIs for PD improved QoL and comorbid depressive symptoms. Of note, however, in the current study, the QoL effect was specifically found for psychological QoL, while for the remaining QoL domains, no significant effect was observed. Psychological QoL might be the primary domain of improvement as it is the domain most closely related to mental health. This view is supported by our finding that, across all QoL domains, the lowest baseline values were consistently reported in the psychological domain for all groups. Also, a recent validity analysis of the WHO-QOL [55] found that, out of all QoL domains, the psychological domain is the best predictor for overall QoL.

Of note, no significant treatment effects were found for comorbid general anxiety, although in the previous meta-analyses [12,13], similar positive effects of IMIs were found for anxiety as for comorbid depression and QoL. Also, we found no effects for anxiety sensitivity and perceived general self-efficacy. These findings are unexpected, given that CBT interventions, including interventions with interoceptive exposure or symptom provocation, seem to effectively reduce anxiety sensitivity [56] and increases in self-efficacy as well as decreases in anxiety sensitivity appear to predict subsequent symptom severity reductions in CBT for PD with and without agoraphobia [57]. Potential explanations for these findings might, however, be insufficient statistical power to detect such changes in secondary outcomes and divergent mechanisms of change in IMIs compared to classical CBT. Furthermore, the focused nature of the therapeutic material featured in the exposure therapy app might also have reduced its ability to improve anxiety symptoms in a broader sense beyond improvements in core PD with and without agoraphobia symptoms.

### Response and Safety Analyses

Our analyses estimated that approximately 1 in 4 participants achieved substantial improvement in the exposure app group compared to WL and the meditation app, and that rates for reliable improvement and remission were highest in this group. As no adverse events were reported for the exposure app and rates of deterioration were lower or comparable to the other groups, our results indicate that using the exposure app is safe for individuals with PD with and without agoraphobia. In sum, these findings suggest that the exposure app can be a valuable treatment option in addition to established psychological and pharmacological strategies. However, to avoid patient demotivation, clinicians should clearly indicate that, while some patients might greatly benefit from using the exposure app, many patients might also see no relevant change in their symptom severity.

### Limitations and Future Directions

One major limitation of the current study is the high dropout rate, especially the high rate of participants who were lost at follow-up in the exposure app group (43%). This attrition clearly reduced statistical power. The particularly high number of dropouts in the exposure app group might be caused by the study design. After completing the core part of the intervention, some participants in the exposure app group presumably no longer saw any benefit in continuing their study participation. This might be particularly applicable for those participants who responded best, as 3 out of 7 participants who had reached remission according to the PAS at post-assessment subsequently dropped out of the study. Conversely, the WL group exhibited the highest completer rate, as participants in this group might have been highly motivated to complete the study to gain access to the treatment. Future studies should further investigate long-term effects and implement strategies that increase participants’ incentives to complete the study, regardless of their group allocation.

Secondly, overall PD with and without agoraphobia symptom severity at baseline was intermediate in this study according to the classification of Bandelow [8]. In addition, due to the lack of stratified randomization, group differences in baseline symptom severity were observed in that the meditation group had lower overall PD with and without agoraphobia symptom scores than the other groups. While we controlled for symptom severity at baseline in our statistical models, it is still possible that treatment effects of both the exposure therapy app as well as the meditation app to some degree depend on the symptom severity level. For example, some types of exercises, such as longer meditations or longer in-vivo exposure sessions, might be difficult for participants with very severe symptoms. Therefore, further studies of relatively small or moderate sample sizes should use a stratified randomization procedure and explore the effects of app-guided exposure therapy systematically in samples with high and low symptom severity.

A further caveat of the current study is the relatively short intervention period of 5 weeks. Previous RCTs on IMIs for PD with and without agoraphobia featured in the meta-analysis of Polak et al [13] typically used treatment periods ranging from 6 to 12 weeks. This may also be reflected in the delayed treatment effects for our secondary outcomes, which only became apparent at follow-up, but not immediately at post-treatment. Therefore, the intervention period of this study might not have been sufficient to reveal all potential effects, and a longer intervention period could possibly lead to stronger post-treatment effects in future studies.

An additional limitation of our study is that we could not guarantee that participants were kept completely blind, as information documents about the study stating that the exposure therapy intervention was the intervention of interest had to be made available online (eg, in the study registration) and had to be disclosed to obtain informed consent.

Another important note about the current study is that a large part of data collection took place during Covid-19 restrictions, including temporary lockdowns. This might have seriously restricted the realization and effectiveness of in-vivo exposure exercises, especially exercises that required using public transport and being in crowded places. Hence, it is conceivable that the effects found in the exposure app group would have been stronger if there had been no Covid-19 restrictions and exposure exercises could have been carried out more easily.

More generally, future research should attempt to systematically investigate where, when, and how participants used the exposure therapy app, in order to investigate what factors do influence the efficacy of this type of IMI. In addition, the relationship between participant characteristics, such as attitudes to digital technology or previous treatment experiences, and the efficacy of app-guided exposure therapy should be analyzed. A further point to systematically investigate is the optimal level of guidance required for the use of IMIs. In our study, participants received only a brief introduction before beginning the unguided self-help interventions. Of note, however, a recent meta-analysis suggests that guided self-help interventions in PD with and without agoraphobia, ie, self-help interventions with regular therapist contact, are more beneficial than unguided interventions [58]. Hence, while an unguided intervention like the exposure therapy app might offer benefits such as greater cost efficiency compared to guided interventions, user engagement and efficacy might be lower for this type of IMIs. Therefore, future research should also investigate how systematic variation of therapist guidance might influence the efficacy of the here tested app.

### Conclusions

This RCT found evidence for the efficacy of app-guided exposure therapy for PD with and without agoraphobia. The evaluated app improved overall PD with and without agoraphobia symptom severity as well as depressive symptoms and psychological QoL and reduced safety maneuvers. Greater improvement for overall PD with and without agoraphobia symptoms in exposure app versus WL comparisons was found. However, we were unable to demonstrate any superiority of the exposure app compared to our active control condition, an app focused on mindfulness meditation. While descriptively, participants assigned to the exposure app showed greater and more sustained improvements than participants in the meditation app group, these differences were not statistically significant. The extent to which this was due to a lack of statistical power needs to be determined in future large-scale confirmatory studies, since the general feasibility, safety, and efficacy of mobile exposure therapy for PD with and without agoraphobia has been indicated here. Additional directions for future research include investigations aimed at the role of therapist guidance, usage intensity, situational factors of app use, and patient characteristics. The observed response and remission rates indicate that, while some patients might greatly benefit from using the exposure app, many patients might also see no relevant change in their symptom severity. Overall, our findings suggest that app-guided exposure therapy can be a useful further treatment option in addition to established psychological and pharmacological strategies available for PD with and without agoraphobia.

## Supplementary material

10.2196/76389 Multimedia Appendix 1 Table S1: Completers per group and outcome.

10.2196/76389 Multimedia Appendix 2 Table S2: Usage patterns (weekly data).

10.2196/76389 Multimedia Appendix 3 Table S3: Detailed results of our intention-to-treat analysis for primary outcomes.

10.2196/76389 Multimedia Appendix 4 Table S4: Detailed results of our completer analyses for primary outcomes with significant first-stage *F *statistic.

10.2196/76389 Multimedia Appendix 5 Table S5: Detailed results of our intention-to-treat analyses for secondary outcomes with significant first-stage *F *statistic.

10.2196/76389 Checklist 1 CONSORT-EHEALTH checklist.

## Abbreviations

ACQ: Agoraphobic Cognitions Questionnaire
ASI: Anxiety Sensitivity Index
BSQ: Body Sensations Questionnaire
CBT: cognitive behavioral therapy
CONSORT: Consolidated Standards of Reporting Trials
DASS: Depression, Anxiety and Stress Scale
GSES: General Self-Efficacy Scale.
IMIs: internet- or mobile-based interventions
ITT: intention-to-treat
LMM: linear mixed effects models
MI: Mobility Inventory
NNT: number needed to treat
PAS: Panic and Agoraphobia Scale
PD: panic disorders
QoL: quality of life
RCT: randomized controlled trial
TSMS: Texas Safety Maneuver Scale
WHO-QOL: World Health Organization Quality of Life
WL: waiting list
