# Supplementary material for: Mobile App–Guided Exposure Therapy for Panic Disorder With and Without Agoraphobia: Randomized Controlled Trial
Source: J Med Internet Res. 2025 Nov 19;27:e76389. doi: 10.2196/76389 (PMC12629522; doi:10.2196/76389)
Supplement: Multimedia Appendix 1 [file jmir-v27-e76389-s001.pdf]

S1: Completers per group and outcome

	Exposure Therapy			Mindfulness Meditation			Waiting List		
	**T0**	**T1**	**T2**	**T0**	**T1**	**T2**	**T0**	**T1**	**T2**
**PAS**	37	31	18	37	34	29	36	31	32
**ACQ**	37	31	18	37	34	28	36	31	32
**BSQ**	37	31	18	37	34	28	36	31	32
**MI**	37	30	17	37	33	28	36	31	32
**TSMS**	37	31	18	37	34	29	37	31	32
**ASI**	37	32	21	37	34	29	37	31	32
**WHO-QOL**	37	32	21	37	34	29	37	31	32
**DASS**	37	32	21	37	34	29	37	31	32
**GSES**	37	32	21	37	34	29	37	31	32

Abbreviations: PAS = Panic and Agoraphobia Scale, ACQ = Agoraphobic Cognitions Questionnaire, BSQ = Body Sensations Questionnaire, MI = Mobility Inventory, TSMS = Texas Safety Maneuver Scale, ASI = Anxiety Sensitivity Index, WHO-QOL = World Health Organization Quality of Life, DASS = Depression, Anxiety and Stress Scale, GSES = General Self-Efficacy Scale.
