# Supplementary material for: Mobile App–Guided Exposure Therapy for Panic Disorder With and Without Agoraphobia: Randomized Controlled Trial
Source: J Med Internet Res. 2025 Nov 19;27:e76389. doi: 10.2196/76389 (PMC12629522; doi:10.2196/76389)
Supplement: Multimedia Appendix 2 [file jmir-v27-e76389-s002.pdf]

S2 – Usage patterns (weekly data)

Weekly number of app uses

	Daily	Several times per week	Once	Never
**Week 1**				
Exposure App (n=37)	7 (18.9%)	25 (67.6%)	4 (10.8%)	1 (2.7%)
Meditation App (n=37)	13 (35.1%)	18 (48.6%)	4 (10.8%)	2 (5.4%)
**Week 2**				
Exposure App (n=32)	5 (15.6%)	10 (31.3%)	11 (34.4%)	6 (18.7%)
Meditation App (n=34)	10 (29.4%)	15 (44.1%)	5 (14.7%)	4 (11.8%)
**Week 3**				
Exposure App (n=31)	2 (6.5%)	15 (48.4%)	10 (32.3%)	4 (12.9%)
Meditation App (n=32)	8 (25.0%)	14 (43.8%)	8 (25.0%)	2 (6.3%)
**Week 4**				
Exposure App (n=32)	4 (12.5%)	12 (37.5%)	5 (15.6%)	11 (34.4%)
Meditation App (n=31)	9 (29.0%)	12 (38.7%)	7 (22.6%)	3 (9.7%)
**Week 5**				
Exposure App (n=30)	2 (6.7%)	8 (26.7%)	12 (40.0%)	8 (26.7%)
Meditation App (n=29)	8 (27.6%)	9 (31.0%)	9 (31.0%)	3 (10.3%)
**Mean %**				
Exposure App (n=162)	12.0%	42.3%	26.6%	19.1%
Meditation App (n=163)	29.2%	41.2%	20.8%	8.7%

Average duration per use

	> 1 h	30 min - 1 h	15 - 30 min	< 15 min
**Week 1**				
Exposure App (n=37)	1 (2.7%)	6 (16.2%)	22 (59.5%)	8 (21.6%)
Meditation App (n=37)	-	1 (2.7%)	22 (59.5%)	14 (37.8%)
**Week 2**				
Exposure App (n=32)	1 (3.1%)	8 (25.0%)	11 (34.4%)	12 (37.5%)
Meditation App (n=34)	-	5 (14.7%)	11 (32.4%)	18 (52.9%)
**Week 3**				
Exposure App (n=31)	-	2 (6.5%)	10 (32.3%)	19 (61.3%)
Meditation App (n=32)	-	7 (21.8%)	10 (31.3%)	15 (46.9%)
**Week 4**				
Exposure App (n=32)	-	4 (12.5%)	10 (31.3%)	18 (56.3%)
Meditation App (n=31)	-	4 (12.9%)	9 (29.0%)	18 (58.1%)
**Week 5**				
Exposure App (n=30)	1 (3.3%)	2 (6.7%)	5 (16.7%)	22 (73.3%)
Meditation App (n=29)	-	4 (13.7%)	9 (31.0%)	16 (55.2%)
**Mean %**				
Exposure App (n=162)	1.82%	13.4%	34.8%	50.0%
Meditation App (n=163)	0%	13.2%	36.6%	50.2%
