# Supplementary material for: Mobile App–Guided Exposure Therapy for Panic Disorder With and Without Agoraphobia: Randomized Controlled Trial
Source: J Med Internet Res. 2025 Nov 19;27:e76389. doi: 10.2196/76389 (PMC12629522; doi:10.2196/76389)
Supplement: Multimedia Appendix 3 [file jmir-v27-e76389-s003.pdf]

S3: Detailed LMM results of our ITT-analysis for primary outcomes

Outcome	F(Group x Time)	Between-Groups	Within-Group
**PAS**	**F(6,148.48) = 3.00**<br>**p = 0.009, η_p^2 = 0.11**	<u>**T1**</u><br>**Expo.(m = 17.47) vs. Cont. (m = 23.11)**<br>**p = 0.042, d = 0.55 (0.04 – 1.05)**<br>Expo. (m = 17.47) vs. Medit. (m = 18.63)<br>p = 1, d = 0.12 (-0.37 – 0.60)<br>Medit. (m = 18.63) vs. Cont. (m = 23.11)<br>p = 0.138, d = 0.41 (-0.08 – 0.90)	<u>**Expo.**</u><br>**T0-T1 (Δ = 5.90)**<br>**p = < 0.001, d = 0.72 (0.23 – 1.21)**<br>**T0-T2 (Δ = 7.09)**<br>**p = < 0.001, d = 0.90 (0.31 – 1.49)**<br>T1-T2 (Δ = 1.19)<br>p = 1, d = 0.13 (-0.45 – 0.71)
		<u>**T2**</u><br>**Expo. (m = 16.28) vs. Cont. (m = 22.44)**<br>**p = 0.04, d = 0.60 (0.01 – 1.19)**<br>Expo. (m = 16.28) vs. Medit. (m = 18.94)<br>p = 0.841, d = 0.25 (-0.34 – 0.84)<br>Medit. (m = 18.94) vs. Cont. (m = 22.44)<br>p = 0.367, d = 0.32 (-0.19 – 0.82)	<u>Medit.</u><br>T0-T1 (Δ = 1.03)<br>p = 1, d = 0.10 (-0.37 – 0.57)<br>T0-T2 (Δ = 0.72)<br>p = 1, d = 0.07 (-0.42 – 0.55)<br>T1-T2 (Δ = -0.31)<br>p = 1, d = -0.03 (-0.52 – 0.47)
			<u>Cont.</u><br>T0-T1 (Δ = 1.17)<br>p = 1, d = 0.12 (-0.37 – 0.60)<br>T0-T2 (Δ = 1.84)<br>p = 0.54, d = 0.19 (-0.29 – 0.66)<br>T1-T2 (Δ = 0.67)<br>p = 1, d = 0.06 (-0.43 – 0.55)
**ACQ**	F(6,141.33) = 1.62<br>p = 0.147, η_p^2 = 0.06		
**BSQ**	F(6,147.08) = 0.79<br>p = 0.582, η_p^2 = 0.03		
**MI accom.**	F(6,138.26) = 0.65<br>p = 0.69, η_p^2 = 0.03		
**MI alone**	F(6,137.85) = 1.78<br>p = 0.108, η_p^2 = 0.07		
**TSMS**	**F(6,142.69) = 2.88**<br>**p = 0.011, η_p^2 = 0.11**	<u>T1</u><br>Expo. (m = 100.27) vs. Cont. (m = 99.53)<br>p = 1, d = -0.02 (-0.52 – 0.48)<br>Expo. (m = 100.27) vs. Medit. (m = 95.48)<br>p = 1, d = -0.11 (-0.60 – 0.38)<br>Medit. (m = 95.48) vs. Cont. (m = 99.53)<br>p = 1, d = 0.08 (-0.40 – 0.57)	<u>**Expo.**</u><br>**T0-T1 (Δ = 12.75)**<br>**p = 0.02, d = 0.32 (-0.17 – 0.79)**<br>**T0-T2 (Δ = 26.66)**<br>**p = < 0.001, d = 0.62 (0.04 – 1.19)**<br>T1-T2 (Δ = 13.91)<br>p = 0.052, d = 0.35 (-0.24 – 0.94)
		<u>T2</u><br>Expo. (m = 86.36) vs. Cont. (m = 102.26)<br>p = 0.394, d = 0.35 (-0.23 – 0.93)<br>Expo. (m = 86.36) vs. Medit. (m = 99.17)<br>p = 0.654, d = 0.29 (-0.31 – 0.88)<br>Medit. (m = 99.17) vs. Cont. (m = 102.26)<br>p = 1, d = 0.07 (-0.44 – 0.57)	<u>Medit.</u><br>T0-T1 (Δ = 3.97)<br>p = 1, d = 0.09 (-0.38 – 0.55)<br>T0-T2 (Δ = 0.27)<br>p = 1, d = 0.01 (-0.48 – 0.49)<br>T1-T2 (Δ = -3.69)<br>p = 1, d = -0.08 (-0.57 – 0.42)
			<u>Cont.</u><br>T0-T1 (Δ = 2.07)<br>p = 1, d = 0.05 (-0.43 – 0.52)<br>T0-T2 (Δ = -0.66)<br>p = 1, d = -0.02 (-0.49 – 0.46)<br>T1-T2 (Δ = -2.73)<br>p = 1, d = -0.06 (-0.55 – 0.44)

Abbreviations: LMM = linear mixed effects models, ITT = intent-to-treat, df = degrees of freedom, η_p^2 = partial eta squared, d = Cohen's d effect size, PAS = Panic and Agoraphobia Scale, ACQ = Agoraphobic Cognitions Questionnaire, BSQ = Body Sensations Questionnaire, MI = Mobility Inventory, TSMS = Texas Safety Maneuver Scale.
