# Supplementary material for: Mobile App–Guided Exposure Therapy for Panic Disorder With and Without Agoraphobia: Randomized Controlled Trial
Source: J Med Internet Res. 2025 Nov 19;27:e76389. doi: 10.2196/76389 (PMC12629522; doi:10.2196/76389)
Supplement: Multimedia Appendix 4 [file jmir-v27-e76389-s004.pdf]

S4: LMM results of our completer analyses for primary outcomes with significant first stage F-statistic

Outcome	F(Group x Time)	Between-Groups	Within-Group
**PAS Completers**	**F(6,91.90) = 2.69**<br>**p = 0.019, η_p^2 = 0.15**	T1<br>Expo.(m = 16.19) vs. WL (m = 22.38)<br>p = 0.166, d = 0.60 (-0.07 – 1.25)<br>Expo. (m = 16.19) vs. Medit. (m = 20.27)<br>p = 0.591, d = 0.40 (-0.26 – 1.06)<br>Medit. (m = 20.27) vs. WL (m = 22.38)<br>p = 1, d = 0.19 (-0.35 – 0.74)	**Expo.**<br>**T0-T1 (Δ = 6.64)**<br>**p = 0.002, d = 0.79 (0.01 – 1.55)**<br>**T0-T2 (Δ = 6.93)**<br>**p = 0.001, d = 0.84 (0.06 – 1.61)**<br>T1-T2 (Δ = 0.29)<br>p = 1, d = 0.03 (-0.71 – 0.77)
		T2<br>Expo. (m = 15.90) vs. WL (m = 22.05)<br>p = 0.171, d = 0.59 (-0.07 – 1.24)<br>Expo. (m = 15.90) vs. Medit. (m = 20.23)<br>p = 0.515, d = 0.41 (-0.25 – 1.07)<br>Medit. (m = 20.23) vs. WL (m = 22.05)<br>p = 1, d = 0.16 (-0.39 – 0.70)	Medit.<br>T0-T1 (Δ = -0.76)<br>p = 1, d = -0.07 (-0.63 – 0.48)<br>T0-T2 (Δ = -0.72)<br>p = 1, d = -0.07 (-0.62 – 0.49)<br>T1-T2 (Δ = 0.04)<br>p = 1, d = 0.00 (-0.55 – 0.56)
			WL<br>T0-T1 (Δ = 1.11)<br>p = 1, d = 0.11 (-0.42 – 0.64)<br>T0-T2 (Δ = 1.44)<br>p = 0.878, d = 0.14 (-0.39 – 0.68)<br>T1-T2 (Δ = 0.33)<br>p = 1, d = 0.03 (-0.50 – 0.56)
**TSMS Completers**	**F(6,91.90) = 2.94**<br>**p = 0.011, η_p^2 = 0.16**	T1<br>Expo.(m = 98.65) vs. WL (m = 103.49)<br>p = 1, d = 0.10 (-0.54 – 0.75)<br>Expo. (m = 98.65) vs. Medit. (m = 104.03)<br>p = 1, d = 0.11 (-0.55 – 0.76)<br>Medit. (m = 104.03) vs. WL (m = 103.49)<br>p = 1, d = -0.01 (-0.55 – 0.53)	**Expo.**<br>T0-T1 (Δ = 10.93)<br>p = 0.274, d = 0.24 (-0.51 – 0.98)<br>**T0-T2 (Δ = 25.36)**<br>**p = < 0.001, d = 0.56 (-0.20 – 1.31)**<br>T1-T2 (Δ = 14.43)<br>p = 0.079, d = 0.33 (-0.42 – 1.07)
		T2<br>Expo. (m = 84.22) vs. WL (m = 107.71)<br>p = 0.334, d = 0.53 (-0.13 – 1.18)<br>Expo. (m = 84.22) vs. Medit. (m = 108.91)<br>p = 0.268, d = 0.55 (-0.12 – 1.21)<br>Medit. (m = 108.91) vs. WL (m = 107.71)<br>p = 1, d = -0.03 (-0.57 – 0.52)	Medit.<br>T0-T1 (Δ = 0.00)<br>p = 1, d = 0.00 (-0.55 – 0.55)<br>T0-T2 (Δ = -4.88)<br>p = 0.935, d = -0.11 (-0.66 – 0.45)<br>T1-T2 (Δ = -4.88)<br>p = 0.935, d = -0.10 (-0.65 – 0.46)
			WL<br>T0-T1 (Δ = 0.52)<br>p = 1, d = 0.01 (-0.52 – 0.54)<br>T0-T2 (Δ = -3.70)<br>p = 1, d = -0.08 (-0.62 – 0.45)<br>T1-T2 (Δ = -4.22)<br>p = 1, d = -0.09 (-0.62 – 0.44)

Abbreviations: LMM = linear mixed effects models, WL = waiting list, Expo. = exposure therapy app, Medit. = mindfulness meditation app, df = degrees of freedom, η_p^2 = partial eta squared, m = mean, d = Cohen's d, PAS = Panic and Agoraphobia Scale, TSMS = Texas Safety Maneuver Scale.
