# Supplementary material for: Mobile App–Guided Exposure Therapy for Panic Disorder With and Without Agoraphobia: Randomized Controlled Trial
Source: J Med Internet Res. 2025 Nov 19;27:e76389. doi: 10.2196/76389 (PMC12629522; doi:10.2196/76389)
Supplement: Multimedia Appendix 5 [file jmir-v27-e76389-s005.pdf]

S5: LMM results of our ITT-analyses for secondary outcomes with significant first stage F-statistic

Outcome	F(Group x Time)	Between-Groups	Within-Group
**DASS_D**	**F(6,148.84) = 3.00**<br>**p = 0.009, η_p^2 = 0.11**	T1<br>Expo.(m = 11.78) vs. WL (m = 16.79)<br>p = 0.14, d = 0.46 (-0.04 – 0.96)<br>Expo. (m = 11.78) vs. Medit. (m = 12.24)<br>p = 1.00, d = 0.05 (-0.44 – 0.53)<br>Medit. (m = 12.24) vs. WL (m = 16.79)<br>p = 0.21, d = 0.41 (-0.08 – 0.90)	**Expo.**<br>T0-T1 (Δ = 2.59)<br>p = 0.302, d = 0.25 (-0.22 – 0.73)<br>**T0-T2 (Δ = 5.22)**<br>**p = 0.014, d = 0.48 (-0.07 – 1.02)**<br>T1-T2 (Δ = 2.62)<br>p = 0.466, d = 0.26 (-0.29 – 0.81)
		**T2**<br>**Expo. (m = 9.16) vs. WL (m = 17.41)**<br>**p = 0.007, d = 0.75 (0.18 – 1.31)**<br>Expo. (m = 9.16) vs. Medit. (m = 14.19)<br>p = 0.179, d = 0.42 (-0.15 – 0.99)<br>Medit. (m = 14.19) vs. WL (m = 17.41)<br>p = 0.608, d = 0.27 (-0.23 – 0.78)	Medit.<br>T0-T1 (Δ = -0.55)<br>p = 1, d = -0.06 (-0.52 – 0.41)<br>T0-T2 (Δ = -2.50)<br>p = 0.360, d = -0.23 (-0.72 – 0.26)<br>T1-T2 (Δ = -1.95)<br>p = 0.693, d = -0.17 (-0.67 – 0.32)
			WL<br>T0-T1 (Δ = -1.97)<br>p = 0.626, d = -0.18 (-0.66 – 0.30)<br>T0-T2 (Δ = -2.59)<br>p = 0.286, d = -0.25 (-0.72 – 0.23)<br>T1-T2 (Δ = -0.62)<br>p = 1, d = -0.05 (-0.55 – 0.44)
**WHO_PSY**	**F(6,144.29) = 3.12**<br>**p = 0.007, η_p^2 = 0.11**	T1<br>Expo.(m = 56.84) vs. WL (m = 51.89)<br>p = 0.55, d = 0.28 (-0.21 – 0.78)<br>Expo. (m = 56.84) vs. Medit. (m = 56.54)<br>p = 1.00, d = 0.02 (-0.46 – 0.50)<br>Medit. (m = 56.54) vs. WL (m = 51.89)<br>p = 0.62, d = 0.29 (-0.20 – 0.78)	**Expo.**<br>T0-T1 (Δ = -0.30)<br>p = 1, d = 0.02 (-0.45 – 0.49)<br>**T0-T2 (Δ = -6.36)**<br>**p = 0.006, d = 0.36 (-0.18 – 0.90)**<br>**T1-T2 (Δ = -6.07)**<br>**p = 0.010, d = 0.32 (-0.23 – 0.88)**
		**T2**<br>**Expo. (m = 62.90) vs. WL (m = 51.85)**<br>**p = 0.014, d = 0.63 (0.07 – 1.19)**<br>Expo. (m = 62.90) vs. Medit. (m = 58.44)<br>p = 0.725, d = 0.24 (-0.32 – 0.81)<br>Medit. (m = 58.44) vs. WL (m = 51.85)<br>p = 0.227, d = 0.42 (-0.09 – 0.93)	Medit.<br>T0-T1 (Δ = 1.46)<br>p = 1, d = -0.11 (-0.57 – 0.36)<br>T0-T2 (Δ = -0.44)<br>p = 1, d = 0.03 (-0.45 – 0.52)<br>T1-T2 (Δ = -1.91)<br>p = 0.872, d = 0.12 (-0.37 – 0.62)
			**WL**<br>**T0-T1 (Δ = 4.40)**<br>**p = 0.036, d = -0.26 (-0.74 – 0.22)**<br>**T0-T2 (Δ = 4.43)**<br>**p = 0.031, d = -0.28 (-0.76 – 0.19)**<br>T1-T2 (Δ = 0.04)<br>p = 1, d = 0.00 (-0.50 – 0.49)

Abbreviations: LMM = linear mixed effects models, ITT = intent-to-treat, WL = waiting list, Expo. = exposure therapy app, Medit. = mindfulness meditation app, df = degrees of freedom, η_p^2 = partial eta squared, m = mean, d = Cohen's d, DASS_D = Depression, Anxiety and Stress Scale – Subscale: Depression, WHO-PSY = World Health Organization Quality of Life – Domain: Psychological Quality of Life.
